# Highly Stretchable Non-volatile Nylon Thread Memory

**DOI:** 10.1038/srep24406

**Published:** 2016-04-13

**Authors:** Ting-Kuo Kang

**Affiliations:** 1 Department of Electronic Engineering, Cheng Shiu University, Niaosong Dist., Kaohsiung City 833, Taiwan

## Abstract

Integration of electronic elements into textiles, to afford e-textiles, can provide an ideal platform for the development of lightweight, thin, flexible, and stretchable e-textiles. This approach will enable us to meet the demands of the rapidly growing market of wearable-electronics on arbitrary non-conventional substrates. However the actual integration of the e-textiles that undergo mechanical deformations during both assembly and daily wear or satisfy the requirements of the low-end applications, remains a challenge. Resistive memory elements can also be fabricated onto a nylon thread (NT) for e-textile applications. In this study, a simple dip-and-dry process using graphene-PEDOT:PSS (poly(3,4-ethylenedioxythiophene) polystyrene sulfonate) ink is proposed for the fabrication of a highly stretchable non-volatile NT memory. The NT memory appears to have typical write-once-read-many-times characteristics. The results show that an ON/OFF ratio of approximately 10^3^ is maintained for a retention time of 10^6^ s. Furthermore, a highly stretchable strain and a long-term digital-storage capability of the ON-OFF-ON states are demonstrated in the NT memory. The actual integration of the knitted NT memories into textiles will enable new design possibilities for low-cost and large-area e-textile memory applications.

Electronic textiles, also known as e-textiles, smart textiles, and smart fabrics^1^, are fabrics that contain electronic elements such as power supplies, sensors, display devices, and memories^2^,^3^,^4^,^5^. The integration of electronic elements into textiles, through conventional textile-processing techniques including weaving, knitting, embroidery, and stitching, can provide an ideal platform for the development of lightweight, thin, flexible, and stretchable e-textiles. This will equip us better to meet the demands of the rapidly growing market of wearable-electronics on arbitrary non-conventional substrates and on the human body^6^,^7^. For a simple e-textile system, the electronic elements may include sensors, a data processor, and an information-storage unit. These information-storage devices are important and fundamental elements in modern digital electronic systems. Recent developments in the field of resistive random access memories (RRAMs), which are based on resistive switching behavior, have attracted much attention because of their high response speeds, multibit storage capabilities, and simple conductor/insulator (or semiconductor)/conductor-sandwich structures^8^,^9^,^10^,^11^,^12^,^13^,^14^,^15^,^16^,^17^,^18^,^19^,^20^,^21^. That is, the RRAM is a type of computer memory that works by changing the resistance of materials. When an electric voltage or current is applied to a material, the resistance of the material changes and this resistance can be measured. The operation of the RRAM depends on the principle of resistive hysteresis, which is also the concept behind the ‘memristor’^10^. To date, various insulating or semiconducting materials have been used in resistive memory applications, such as binary transition metal oxides^11^,^12^ or organic materials^8^,^13^,^14^,^15^,^16^,^17^,^18^,^19^,^20^,^21^. For manufacturing flexible and non-volatile memories, which are based on organic materials^15^ and implemented on flexible substrates, poly(3,4-ethylenedioxythiophene) polystyrene sulfonate (PEDOT:PSS which is an organic material) has been found to be suitable because of its low temperature of processing, low cost, and ease of fabrication. Moreover, graphene-based organic hybrid materials have also attracted much attention in the development of the future flexible and stretchable non-volatile memories because of their solution-processability^16^,^17^. The write-once-read-many-times (WORM) memory using the PEDOT:PSS organic material^18^ has been fabricated and demonstrated. The same PEDOT:PSS organic material-based bipolar, unipolar, and nonpolar RRAM devices have also been reported^19^,^20^,^21^. Several examples of polymer-based e-textiles, such as those containing transistors, logic circuits, sensors, antennas, and batteries that are rechargeable by solar energy, on fibers or fabrics^22^,^23^,^24^,^25^,^26^, have been demonstrated. However, the actual integration of these electronic components into textiles that undergo mechanical deformation during assembly and daily wear, or that satisfy the requirements of the low-end applications, still remains a challenge. For example, the e-textile fibers may be exposed to bending radii as small as 160 um during weaving. This corresponds to a tensile bending strain of about 17%^27^; and a maximum strain of about 20% may be present at the shoulders of a shirt^28^. A previous report showed that energy-storing textiles were fabricated using a simple two-step dip-and-dry process that was similar to the dyeing process widely used in the textile industry^29^. Therefore, the simple two-step dip-and-dry process using a solution-processable organic hybrid material^16^,^17^,^30^ is widely believed to be one of the most promising ways to realize truly low-cost and large-area e-textiles through conventional weaving or knitting techniques^31^.

In this study, a highly stretchable nylon thread (NT) memory is fabricated by performing the simple two-step dip-and-dry process using graphene-PEDOT:PSS conductive ink (the details are provided in the Methods section). In the first step, the dipping process, the solution-processable hybrid material of graphene-PEDOT:PSS is adsorbed, and it adheres onto the NT fibers. The second step consists of the drying process in which the residual aqueous solution on the NT microfibers is fully removed to form the NT memory. Through electrical measurements, it can be found that the non-volatile NT memory has typical WORM characteristics. In addition, both high stretchability and long-term digital-storage capability are demonstrated by this NT memory. The actual integration of the non-volatile NT memory into clothes by conventional knitting techniques appears to produce a highly stretchable strain of more than 50%, and it further opens up new design possibilities for future low-cost and large-area e-textile memory applications.

## Results

### Fabricating a stretchable non-volatile NT memory

**Figure 1:**
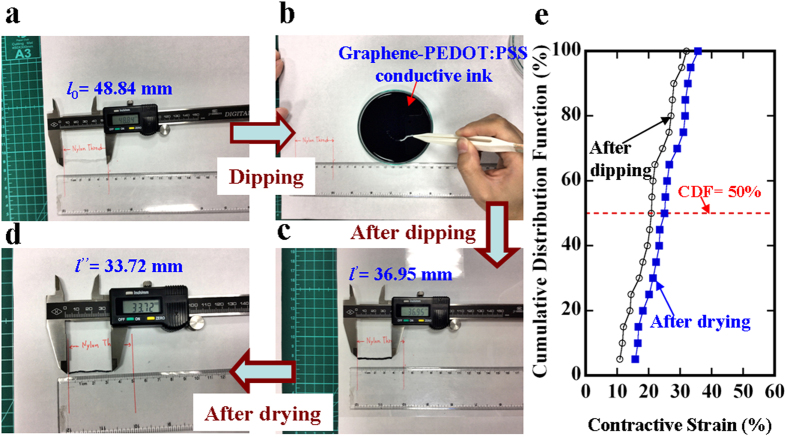
Simple dip-and-dry process employed to fabricate the NT memory. The process is described as follows: (**a**) cut an NT with a fixed length (*l*_0_); (**b**) dip the NT into a graphene-PEDOT:PSS conductive ink; (**c**) the NT has a shorter length (*l*’) compared to the initial fixed length after being dipped; (**d**) form the NT memory after drying at room temperature and in ambient conditions for two days. (**e**) Cumulative distribution function of the two contractive strain values is estimated for 20 samples after being dipped and dried.

Figure 1a–d shows the simple dip-and-dry process, with two steps, for fabricating the NT memory. The elastic NT consists of hundreds of nylon microfibers (Supplementary Fig. S1a). First, an elastic NT of fixed length was dipped into the graphene-PEDOT:PSS conductive ink for 30 min, as shown in Fig. 1a,b. The graphene-PEDOT:PSS conductive ink was prepared using the electrolytic exfoliation of graphite electrodes^32^,^33^. The initial fixed length of the NT decreased after dipping, as shown in Fig. 1c. In the first step, the contractive strain causing the NT shrinkage was due to the densely packed nylon microfibers and the solidification of the PEDOT:PSS because of the aqueous solution loss and the graphene flakes wrapping around the nylon microfibers (Supplementary Fig. S1b). Here, the strain is defined as the change in length (Δ*l*) divided by the initial length (*l*_0_) of the NT. If the NT decreases in length, the strain will be referred to as contractive strain; and if the NT increases in length, then it will be referred to as stretchable strain. To avoid bending the NT, the drying process in the second step was performed at room temperature rather than at high temperatures. To remove all the residual water, the NT that was dipped in the graphene-PEDOT:PSS ink was dried at room temperatures and under ambient conditions for two days, as shown in Fig. 1d. After the drying process, the contractive strain in the second step was measured and it was found that more NT shrinkage had been induced due to the same causes of fully removing the residual aqueous solution and the dense packing of the nylon microfibers. The color of the NT had changed from white to black. The optical and scanning electron microscopy images show a marked decrease in the length of the NT, and in the wrapping of the graphene-PEDOT:PSS sheets around the NT, respectively (Supplementary Fig. S1). The composition of the sheet was confirmed by Raman spectroscopy, and it demonstrated lower defect states in the graphene due to the presence of the D peak and spectral features at the symmetric and asymmetric C_α_ = C_β_ band of the PEDOT (Supplementary Fig. S2). The magnitude of the contractive strain in the NT was obtained using the expression for the normalized change in the length, and it attained the contractive-strain saturation value after approximately 10 h (Supplementary Fig. S1c). Thus, the non-volatile NT memory was fabricated using the simple two-step dip-and-dry process. That is, the non-volatile NT memory is based on the hybrid material of the PEDOT:PSS doped with multilayer graphene flakes. The findings, which showed a G peak with a relatively larger proportion than the 2D peak, were an indication of the presence of multilayer graphene flakes (Supplementary Fig. S2). The cumulative distribution function of two contractive strain values in the NT memory was estimated using 20 samples after the dipping and drying processes, as shown in Fig. 1e. For the first and second step of the simple dip-and-dry process, the two average contractive strain values of approximately 21% and 25% further suggest that a highly stretchable non-volatile NT memory can be formed.

### WORM characteristics

**Figure 2:**
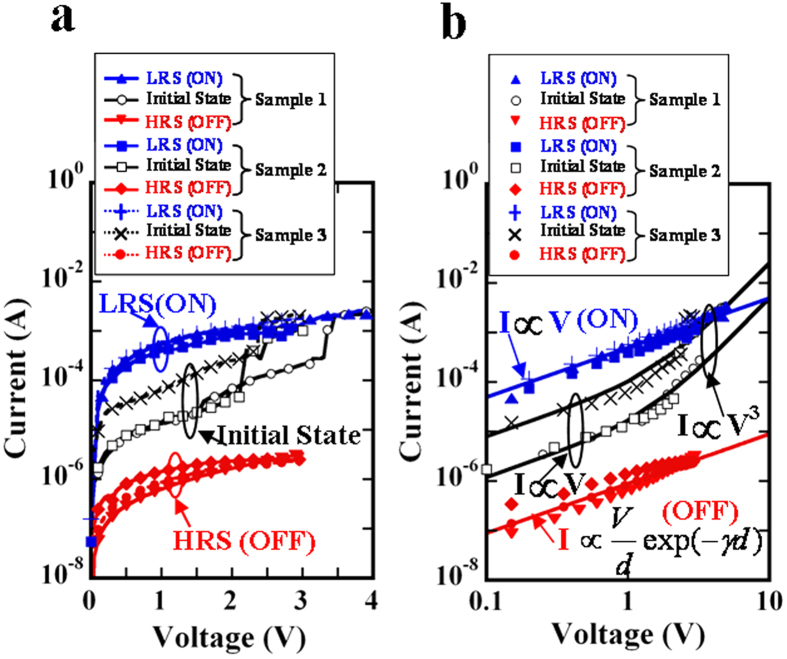
Electrical characteristics of the NT memory. (**a**) I-V characteristics of the NT memory with a length of 3 cm appearing in three states. (**b**) Log-log scale and fitting lines of the I-V data are plotted and calculated to understand the possible mechanisms.

Figure 2a shows the current-voltage (I-V) characteristics of the non-volatile NT memory, which contains three states: initial state, low-resistance state (LRS), and high-resistance state (HRS). First, all the virgin NT memories were found to be almost in the initial state. The initial state was defined as a resistance value without applied strains. When the applied voltage was swept from 0 to 4 V, there was a marked increase in the current. The initial state of the NT memory was switched to the LRS, which is denoted as the ON state. This switching behavior from the initial state to the LRS is called the forming process. When the applied voltage was swept in the opposite direction, from 4 to 0 V, the NT memory was switched possibly from the LRS to the initial state. A pure PEDOT:PSS NT memory was also fabricated using the same two-step dip-and-dry process. Compared to the NT memory dipped in the graphene-PEDOT:PSS ink, the forming process was not observed in the pure PEDOT:PSS NT memory (Supplementary Fig. S3). This implies that the multilayer graphene flakes randomly distributed in the PEDOT:PSS may act as charge-trapping sites^34^. In addition, more NT memory samples in Fig. 2a have demonstrated the resistive switching behavior from the initial state to the LRS, i.e., the forming process. The multilayer graphene flakes acting as charge-trapping sites explain this forming process. After sweeping one or two cycles (from 0 to 4 V and 4 to 0 V), the NT memory can remain in the LRS due to the formation of conductive filaments and there appears to be a stable ON state. No marked difference has occurred yet in the I-V characteristics, between the samples in the ON state. Similar resistive switching behaviors were observed for the negative voltage sweep (Supplementary Fig. S4). Then, by sweeping a high voltage from 3 to 10 V, the NT memory was switched from the LRS to the HRS. It was shown to be in a stable HRS, denoted as the OFF state. This switching behavior from the LRS to the HRS is called the writing process. Even though the applied voltage was swept again from 0 to 10 V, the NT memory remained in the HRS (OFF) and appeared to have typical WORM characteristics. This implies that the permanent HRS can be attributed to the phase-segregation mechanism of resistive switching in the PEDOT:PSS, which is caused by the high switching current^18^,^35^. To understand the resistive switching mechanism in a better manner, the previous I-V curves were plotted in a log-log scale, as shown in Fig. 2b. These I-V characteristics for the initial state with three different samples at low voltages, and at the LRS (ON), can be explained by ohmic conduction: I  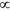 V. Furthermore, the LRS (ON) obeys the well-known Ohm’s law, further providing evidence for the formation of conductive filaments. Similarly, for the initial state with three NT memory samples measured at high voltages, the I-V conduction can be attributed to a trap-controlled space-charge-limited current, I  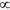 V^3^, which is due to the charge trapping in the multilayer graphene flakes^34^. Then, the HRS current dominated by the insulating PSS in the PEDOT:PSS can be described using the following tunneling model^36^,^37^:


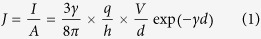


where A is the area of cross-section of the NT memory, q is the electronic charge, h is Planck’s constant, V is the applied voltage, d is the tunneling distance, and γ is defined as


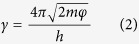


where m and φ are the carrier mass and the potential barrier height, respectively. A was determined to be approximately 3.4 × 10^10 ^nm^2^ by reading the diameter of the NT memory. According to equations (1) and (2), the tunneling model can be determined by adjusting the two fitting parameters of φ = 1.0 eV and d = 6.78 nm to obtain a reasonable agreement with the HRS currents of two different samples (Fig. 2b). In addition, the discrepancy in the HRS currents of the tunneling model and sample 2 can be explained by adjusting the fitting parameters to either φ = 0.9 eV or d = 6.74 nm. This further indicates that for different NT memory samples, the variance in the HRS currents is mainly attributed to the change in the quantity of the insulating PSS in the PEDOT:PSS.

### Evaluating the retention time and the stretching capability

**Figure 3:**
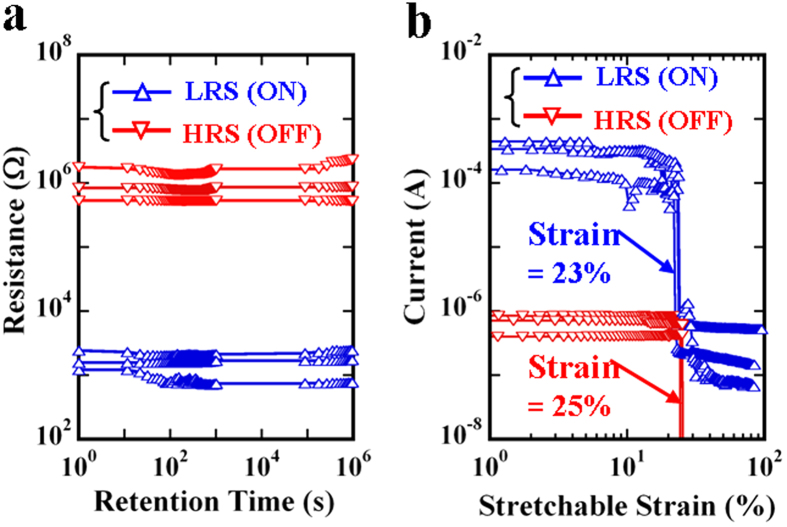
Retention time and mechanical stretching measurements. (**a**) Retention time of the LRS and HRS at a reading voltage of 0.5 V appears to be more than 10^6 ^s. A marked difference between the LRS and HRS is shown to be approximately 10^3^. (**b**) LRS and HRS currents are measured at a reading voltage of 0.5 V, and are simultaneously stretched by manually moving one mini vise to evaluate the stretching capability of the NT memory. Two average stretchable strain values of approximately 23% and 25% are shown for the LRS and HRS, respectively.

Figure 3a,b demonstrates the evaluation of the retention time and the stretching capability of the non-volatile NT memory, respectively. During retention testing with three samples, an LRS:HRS ratio of approximately 10^3^ was maintained for a retention time of 10^6 ^s at a reading voltage of 0.5 V, as shown in Fig. 3a. This result further supports the fact that different NT memory samples still have retention times of 10^6 ^s under an LRS:HRS ratio of about 10^3^. Furthermore, based on the length reading with a high-resolution digital caliper, the stretchable strain could be accurately obtained by the expression: 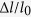, where *l*_0_ is the initial length of the NT memory and Δ*l* is defined as the elongation in the stretching NT memory when there is an abrupt decrease in the LRS and HRS currents. Figure 3b shows the same number of NT memory samples (three) being used to evaluate the stretching capabilities by manually moving one mini vise. Even though the moving speed appears to change slightly from 0.17 to 0.26 mm/s (see the Methods section), no significant variations are found in the stretchable strain, regardless of the LRS and HRS, as shown in Fig. 3b. The results of the three different samples further indicate that slight changes in the moving speed can be neglected for the determination of the stretchable strain. Then, two average values (of the stretchable strain) of approximately 23% and 25% were found for the LRS and HRS of the three different samples, respectively. While stretching the NT memory at a low moving speed, a detailed record of the LRS and HRS currents as a function of time at a reading voltage of 0.5 V was measured (Supplementary Fig. S5) and recorded in videos (see Supplementary videos). Typical stress-strain curves were also evaluated to obtain an ultimate stretchable strain of about 39% for the NT memory, at a maximum stretchable stress of about 3.3 MPa (Supplementary Fig. S6). A stretchable strain larger than 20% can be attributed mainly to an initial average contractive strain of approximately 25% in the NT memory, caused by the simple dip-and-dry process, which is similar to the pre-strained elastomer effect^38^.

### A highly stretchable NT memory

**Figure 4:**
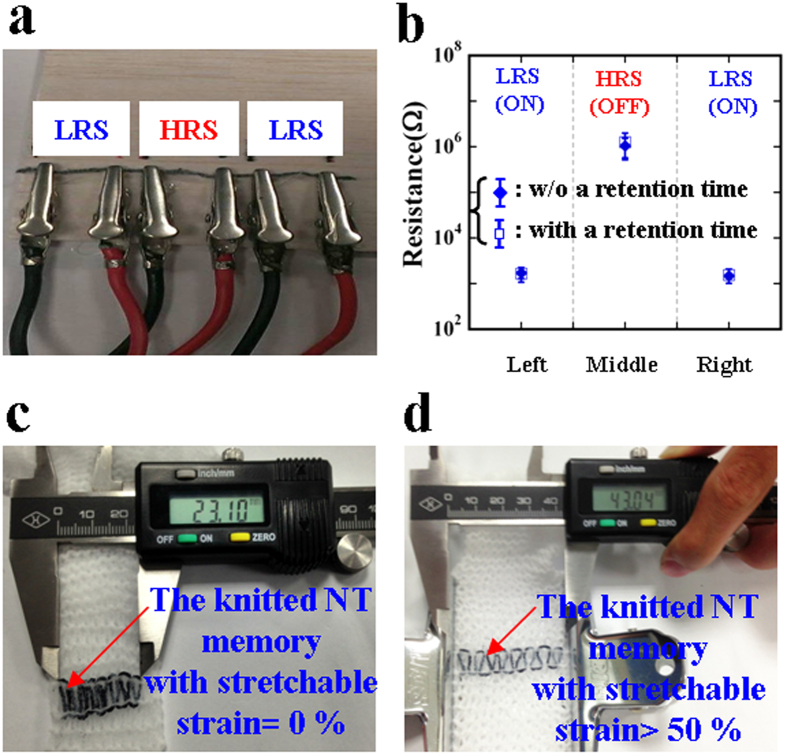
Evaluating the long-term digital-storage and highly stretchable capabilities. (**a**) Electrical measurement setup for the NT memory having three segmented lengths with LRS, HRS, and LRS behavior, in that order. (**b**) Measured resistances of the three segmented lengths to evaluate the retention capability of the NT memory. The square and diamond symbols represent a comparison of these resistances in the NT memory with and without testing, respectively, for a retention time of 10^6 ^s. (**c**) and (**d**) show the knitted NT memory integrated into a textile with stretchable strain = 0% and stretchable strain >50%, respectively.

The non-volatile NT resistive memory has three segmented lengths (Fig. 4a) with LRS, HRS, and LRS behavior, in that order, and which appears to be the long-term digital-storage capability of the ON-OFF-ON states (Fig. 4b). The stored LRS in each segmented length can be obtained using a pair of alligator clips connected to the NT memory, by sweeping a positive voltage from 0 to 4 V. Subsequently, the stored LRS in the middle segmented length can be switched to the stable HRS by sweeping a high voltage from 3 to 10 V. Similarly, to evaluate the retention capability using the digital storage of LRS-HRS-LRS, these currents with time are also read at a voltage of 0.5 V. After retention testing with three NT memory samples, little difference was observed in these resistances both with and without the retention time of 10^6 ^s for each segmented memorized length. In Fig. 4b, the error bar graph of the statistic distribution from three different NT memory samples verifies that the retention capability of the digital storage of the LRS-HRS-LRS is stable with a long-term retention time of 10^6 ^s under an LRS:HRS ratio of approximately 10^3^. In addition, the non-volatile NT memory with the digital storage of LRS-HRS-LRS (i.e., ON-OFF-ON states) integrated into textiles by a conventional knitting machine demonstrates an interlock-knitted structure^39^ and a high stretchable strain of larger than 50%, as shown in Fig. 4c,d.

## Discussion

In this study, 20 samples were prepared to provide information about the statistic distribution of the contractive strain existing in the NT memory. Owing to the simple two-step dip-and-dry process, there was an initial average contractive strain of approximately 25% in the NT memory (Fig. 1e), which was similar to the pre-strained effect^38^ on the NT memory. The pre-strained effect can further support the fact that the non-volatile NT memory has a high stretchable strain of approximately 23%. The absence of any marked difference between the calculation of the tunneling model and the HRS current (Fig. 2b) verifies the existence of stable insulating PSS barriers caused by the current-induced PEDOT:PSS phase segregation, clearly showing the write-once HRS behavior. Thus, the non-volatile NT memory appears to have typical WORM characteristics. According to previous studies regarding the writing process caused by the phase-segregation mechanism^18^,^35^, the resistive increase by up to six orders of magnitude remains stable for at least one month, further supporting our retention result. That is, an ON/OFF ratio of approximately 10^3^ is maintained for a retention time of 10^6 ^s (Fig. 3a). Even though more NT memory samples have been measured, significant degradation in the ON/OFF ratios with the retention time of 10^6 ^s has not yet been found. This further implies that the retention time of at least one month is predictable. Moreover, after the retention time of 10^6 ^s, only the ON state can be reprogrammed to the OFF state, and the OFF state is shown to be irreversible. The long-term digital-storage capability of the ON-OFF-ON memorized behavior can also be demonstrated in the non-volatile NT memory fabricated using the same two-step dip-and-dry process. Finally, through a conventional knitting technique, the actual integration of the highly stretchable non-volatile NT memory into clothes (Fig. 4c,d) will lead to new design possibilities for low-cost and large-area e-textile memory applications. At present, some issues concerning the product development of the proposed non-volatile NT memories, (regarding the thermal stability, humidity, and washability) certainly need further research.

## Methods

### Graphene-PEDOT:PSS ink

The ink was purchased from the Innophene Company Limited, Thailand, and it was synthesized using the electrolytic exfoliation of graphite^32^,^33^. The electrolytic exfoliation method used two graphite rods placed in an electrolytic cell with a liquid PEDOT:PSS electrolyte. When a constant potential was applied between the graphite electrodes, the positive electrode started corroding and a black precipitate was gradually formed in the electrolysis cell. The exfoliation process continued for several hours to obtain a suitable graphene concentration dispersed in the liquid PEDOT:PSS electrolyte. The dispersed graphene-PEDOT:PSS solution, which was used as ink, was taken from the electrolysis cell and was centrifuged at a low speed to filter out the large agglomerates. Finally, the spectral features of the graphene-PEDOT:PSS sheets were observed using the micro-Raman system (LabRAM HR, Horiba Jobin Yvon) with an excitation energy of 2.33 eV (Laser 532 nm).

### Electrical and mechanical stretching measurements

All the electrical characteristics of the non-volatile NT memory were measured at room temperature with a Keithley 2400 source meter. Based on the reading obtained with a digital caliper having a resolution of 0.01 mm, the elongation of the stretchable NT memory was utilized to evaluate the stretching capability. Finally, the stretching capability was obtained by manually moving one mini vise at a low speed of approximately 0.2 mm/s (see Supplementary videos). In this study, the moving speed of 0.2 mm/s was chosen as the average of the moving speeds ranging from 0.17 to 0.26 mm/s, and was defined as the ratio of the elongation in the NT memory to the stretching time. The stretching time can be measured using an electronic timer, which records the difference in time between the start and end of the NT memory stretching. The electronic timer recorded the time until there was an abrupt decrease in the LRS or HRS current on stretching the NT memory.

## Additional Information

**How to cite this article**: Kang, T.-K. Highly Stretchable Non-volatile Nylon Thread Memory. *Sci. Rep.* **6**, 24406; doi: 10.1038/srep24406 (2016).

## Supplementary Material

Supplementary Information

Supplementary Video 1

Supplementary Video 2
