# Supplementary material for: Highly Stretchable Non-volatile Nylon Thread Memory
Source: Sci Rep. 2016 Apr 13;6:24406. doi: 10.1038/srep24406 (PMC4829934; doi:10.1038/srep24406)
Supplement: Supplementary Information [file srep24406-s1.pdf]

Supplementary Information

Highly Stretchable Non-volatile Nylon Thread Memory

Ting-Kuo Kang

Supplementary Figure S1. (a) and (b) show two optical image cross-sections of the NT with and without the application of the dip-and-dry process, respectively. (c) After the simple dip-and-dry process, the evolution of the contractive strain is measured, and attains a saturation value after approximately 10 h. (d) Scanning electron microscopy image shows the wrapping of the graphene:PEDOT:PSS sheets around the NT (scale bar = 500 um); (e) Magnified image of the sheets (scale bar = 200 um).

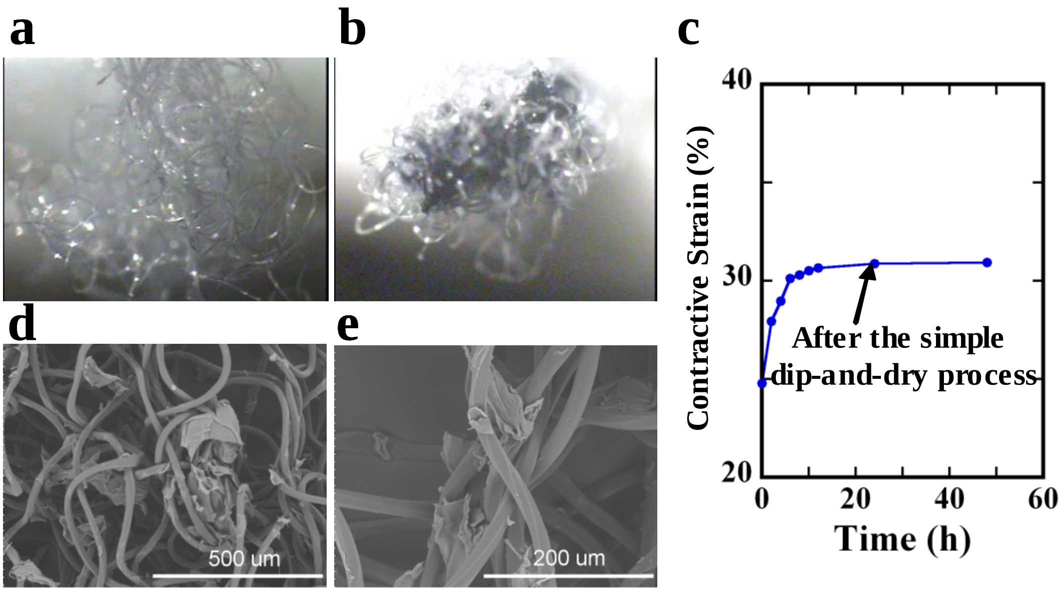

Supplementary Figure S2. Raman spectra of the graphene-PEDOT:PSS sheets and the graphene powders extracted from the conductive ink. The spectral features are confirmed and indicated with dotted lines, further demonstrating that the graphene has lower defect states due to the presence of the D peak at ~ 1330 cm^{-1} [S1]. Peak positions of the symmetric and asymmetric C_α=C_β band of the PEDOT are observed[S2]. The 2D peak at ~ 2670 cm^{-1} less than the G peak is also shown for the confirmation of the presence of multilayer graphene flakes[S3].

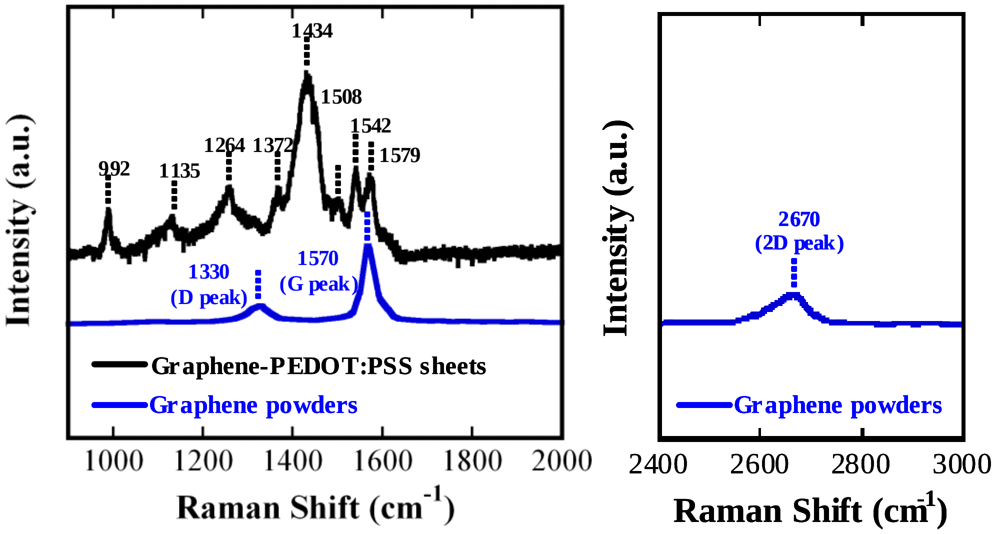

Supplementary Figure S3. I-V characteristics of the pure PEDOT:PSS NT memory samples appearing in only two states of LRS (ON) and HRS (OFF).

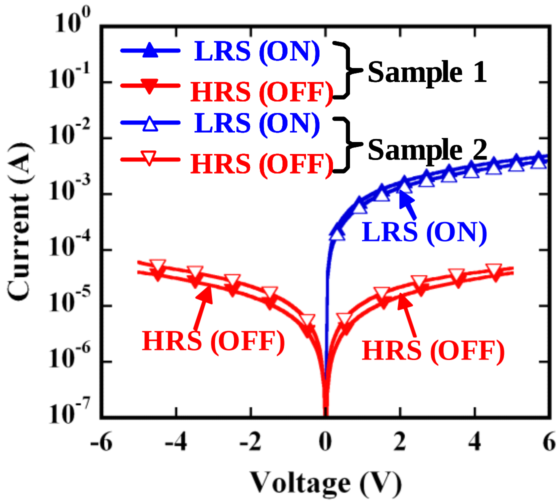

Supplementary Figure S4. (a) Typical I-V curves of the NT memory with a length of 2 cm for negative voltage sweep. (b) High current obtained by sweeping a high voltage from 3 to 10 V drives the NT memory switching from the LRS to HRS, further demonstrating the write-once HRS behavior.

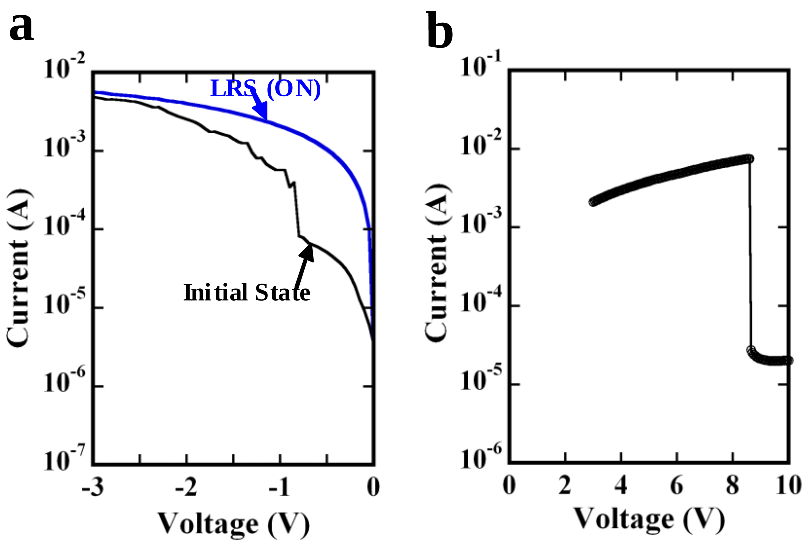

Supplementary Figure S5 and videos of stretchable LRS and HRS

While stretching the NT memory, detailed progression of the LRS (ON) and HRS (OFF) currents versus time at a reading voltage of 0.5 V is shown in Fig. S5 and recorded in two video files (Stretchable LRS and Stretchable HRS). The video has two graphs.

The stretchable LRS video displays a subtitle of LRS and a description of the two graphs. The description is that top and bottom graphs show a reading voltage of 0.5 V and the measured currents with time, respectively.

The stretchable HRS video displays a subtitle of HRS and a description of the two graphs. The description is that top and bottom graphs show a reading voltage of 0.5 V and the measured currents with time, respectively.

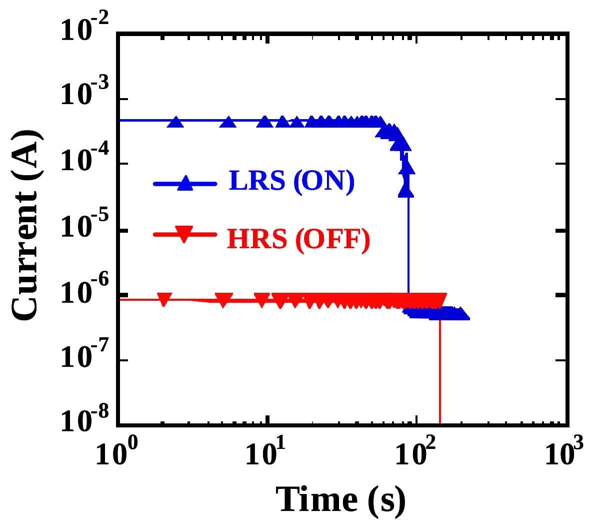

Supplementary Figure S6. Typical stress versus strain curves used to evaluate the ultimate stretchable strain. The stress is defined as F/A, where F is the applied force and A is the area of cross-section. The magnitude of the applied force can be determined by a pull dynamometer. The average values of the ultimate stretchable strain and the maximum stretchable stress are found to be approximately 39% and 3.3 MPa, respectively.

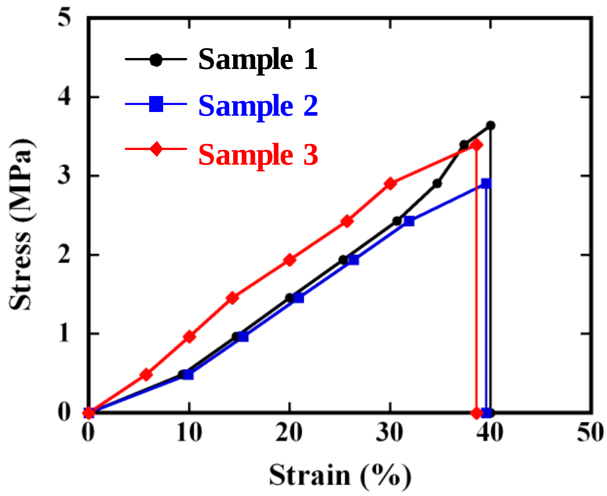

References

S1 Pimenta, M. A. et al. Studying disorder in graphite-based systems by Raman spectroscopy. *Phys. Chem. Chem. Phys.* **9**, 1276-1290 (2007).

S2 Garreau, S., Louarn, G., Buisson, J. P., Froyer, G. & Lefrant, S. In situ spectroelectrochemical Raman studies of poly (3,4-ethylenedioxythiophene) (PEDT). *Macromolecules* **32**, 6807-6812 (1999).

S3 Li, X. et al. Large-area synthesis of high-quality and uniform graphene films on copper foils. *Science* **324**, 1312-1314 (2009).
